# Light exposure behaviors predict mood, memory and sleep quality

**DOI:** 10.1038/s41598-023-39636-y

**Published:** 2023-08-01

**Authors:** Mushfiqul Anwar Siraji, Manuel Spitschan, Vineetha Kalavally, Shamsul Haque

**Affiliations:** 1 Department of Psychology, Jeffrey Cheah School of Medicine and Health Sciences and Intelligent Lighting Laboratory, Monash University Malaysia, Jalan Lagoon Selatan, 47500 Bandar Sunway, Selangor Darul Ehsan Malaysia; 2 Max Planck Institute for Biological Cybernetics, Translational Sensory & Circadian Neuroscience, Tübingen, Germany; 3 Department of Sport and Health Sciences (TUM SG), Technical University of Munich, Munich, Germany; 4 Department of Electrical and Computer Systems Engineering and Intelligent Lighting Laboratory, Monash University Malaysia, Jalan Lagoon Selatan, 47500 Bandar Sunway, Selangor Darul Ehsan Malaysia

**Keywords:** Psychology, Environmental sciences

## Abstract

Ample research has shown that light influences our emotions, cognition, and sleep quality. However, little work has examined whether different light exposure-related behaviors, such as daytime exposure to electric light and nighttime usage of gadgets, especially before sleep, influence sleep quality and cognition. Three-hundred-and-one Malaysian adults (Mean_Age±SD_ = 28 ± 9) completed the Light Exposure Behavior Assessment tool that measured five light exposure behaviors. They also completed the Morningness-Eveningness Questionnaire, Positive and Negative Affect Schedule, Pittsburgh Sleep Quality Index, and single items assessing trouble in memory and concentration. A partial least square structural equation model, showing 72.72% predictive power, revealed that less use of wearable blue filters outdoors during the day and more within one hour before sleep predicted early peak time (direct effect = −0.25). Increased time spent outdoors predicted a positive affect (direct effect = 0.33) and a circadian phase advancement (direct effect: rising time = 0.14, peak time = 0.20, retiring time = 0.17). Increased use of mobile phone before sleep predicted a circadian phase delay (direct effect: retiring time = −0.25; rising time = −0.23; peak time = −0.22; morning affect = −0.12), reduced sleep quality (direct effect = 0.13), and increased trouble in memory and concentration (total effect = 0.20 and 0.23, respectively). Increased use of tunable, LED, or dawn-simulating electric light in the morning and daytime predicted a circadian phase advancement (direct effect: peak time = 0.15, morning affect = 0.14, retiring time = 0.15) and good sleep quality (direct effect = −0.16). The results provide valuable insights into developing a healthy light diet to promote health and wellness.

## Introduction

Scientific evidence published over the last four decades has shown that retinal light exposure influences our physiology, behavior, and emotion. More specifically, it modulates human sleep, circadian rhythms, alertness, mood, neuroendocrine and neurobehavioral functions^1–5^. These influences of light on human physiology and behaviors are collectively known as non-image-forming responses (NIF) of light. The melanopsin-enriched intrinsically photoreceptive retinal ganglion cells (ipRGCs), sensitive to short wavelength-enriched (blue-enriched, ~ 480 nm) light^6^, generally mediate the NIF effects of light.

### Light’s influence on chronotype, sleep quality and mood

With the advent of artificial light and self-luminous displays, our retinal light exposure is no more limited to the natural day-night cycle. An extensive body of research suggests that the imbalance of light and dark exposure disrupts our circadian system^7^. Subsequently, this disruption gives rise to a series of adverse consequences, including decreased sleep quality, mood, and an alteration of sleeping habits^7–9^. Since the natural light–dark cycle is the most vital zeitgeber to synchronize our body clock to the astronomical day, altering this cycle forces us to have different chronotype-disposition for activity early or late in the day^10^. Research shows that exposure to bright light (~ 5000–10,000 lux) at night results in a phase delay^11^, and exposure to bright light in the morning leads to a phase advance^12,13^. Increased nighttime light exposure is also associated with decreased sleep quality^14,15^. However, several studies have reported better nighttime sleep quality after exposure to electric light (300–1000 lux) in the morning^9,16–18^. He et al.^17^ observed a higher nocturnal sleep efficiency, earlier sleep onset, shorter sleep latency, and lower morning sleepiness among college students (*N* = 12) when they are exposed to bright light (1000 lux, 6500 K) in the morning for five days compared to conventional office light (300 lux, 4000 K). Brain regions such as limbic areas and the hypothalamic–pituitary–adrenal axis responsible for regulating mood are susceptible to circadian regulation^19^. Thus, it is reasonable to anticipate that the disruption of circadian regulation will disrupt mood regulation^19^. Bright light exposure in the morning increases positive mood; however, exposure to bright light in the afternoon enhances negative mood^20–23^.

### Light exposure, memory, and concentration

Several studies have confirmed that retinal light exposure activates the hippocampus, which is closely associated with memory functions^24–26^. Thus, researchers anticipate that retinal light exposure would influence memory. Vandewalle et al.^27^ observed that, compared to 18 min of exposure to a monochromatic green light (550 nm; 3 × 10^13^ photons/cm^2^/s), 18 min of exposure to a monochromatic blue light (470 nm; 3 × 10^13^ photons/cm^2^/s) improves working memory performance (*N* = 18). Alkozei et al.^28^ reported enhanced verbal memory for a 30-min monochromatic blue light exposure (469 nm; 214 lux; *N* = 12) compared to monochromatic amber light (578 nm; 188 lux). Huiberts et al.^29^ offered further evidence that light influences memory-based task performances, whereby participants performed better in simple than complex tasks under polychromatic white bright light conditions (200 lux, 4000 K vs. 1000 lux, 4000 K; *N* = 64). Retinal light exposure is also associated with improved concentration. Kretschmer et al.^30^ observed an improved concentration among night shift workers (*N* = 32) under a bright light condition (3269–3684 lux vs. 257–339 lux). Sleegers et al.^31^, in their studies on the effects of light in classroom environments, concluded a beneficial influence of a dynamic light environment on students’ concentration (1000 lux, 6500 K vs. 300 lux, 3000–4000 K; *N* = 181).

### Interrelation of chronotype, mood, sleep quality, memory and concentration

Due to social jetlag (misaligned sleep–wake pattern with work schedule), different chronotypes, especially early and late chronotypes, might exhibit a reduced sleep quality. Juda et al.^32^ found that workers with early chronotypes had shorter sleep duration and more sleep disturbances than late chronotypes (*N* = 371 shift workers). Moreover, late chronotypes had poor sleep quality with non-regular sleeping habits during weekdays due to the misalignment of their preferred activity period vs. real-world demands^33–35^. Chronotypes can also influence our memory and concentration^36–38^. Schmidt et al.^36^ reported an interaction effect of chronotype and time of day on memory (*N* = 32). The memory performance of those with early chronotype was better in the morning. In the same vein, the memory performance of those with late chronotype was better in the afternoon^39,40^. Researchers have termed it the *synchrony effect*. Research has also indicated that sleep quality is contingent on mood and vice versa^41,42^. Positive affect- a state of pleasurable engagement with the environment, is associated with improved sleep patterns^43,44^. In contrast, negative affect (feelings of emotional distress) leads to sleep deprivation, poor sleep quality, and reduced cognitive functioning^45–49^. Poor sleep quality, the core symptom of mood disorder, is associated with decreased positive affect^42^. Poor sleep quality also reduces memory functions and concentration^50–54^.

### The current study

Acknowledging the influence of retinal light exposure on our health and well-being, many researchers have attempted to quantify healthy light exposure. They have given recommendations for a healthy indoor light environment that primarily focuses on properties of the light spectrum, such as illuminance and wavelength^55^. However, little effort is visible to study light exposure-related behaviors, which could be an active agent modifying our retinal light exposure. People can control their light exposure through different behaviors by actively seeking or avoiding certain types of light exposure. There is a knowledge gap in understanding these behaviors, which is crucial to developing a healthy light diet- a light exposure pattern promoting health, wellness, and work performance. To address this gap, we have developed the Light Exposure Behavior Assessment (LEBA)^56^ tool, which will facilitate understanding people’s light exposure related behaviors and the development of a healthy light diet. LEBA categorizes five different types of behavior. First, the propensity of wearing blue light filter glasses indoors and outdoors (LEBA B1). Second, the tendency to spend time outdoors (LEBA B2). Third, the usage of mobile phones on the bed before sleeping (LEBA B3). Fourth, our inclination to control environmental light before bedtime (LEBA B4). Finally, the use of electric light (LEBA B5). However, whether these categorizations of behaviors would predict different aspects of our health, memory and concentration remain unknown.

**Figure 1:**
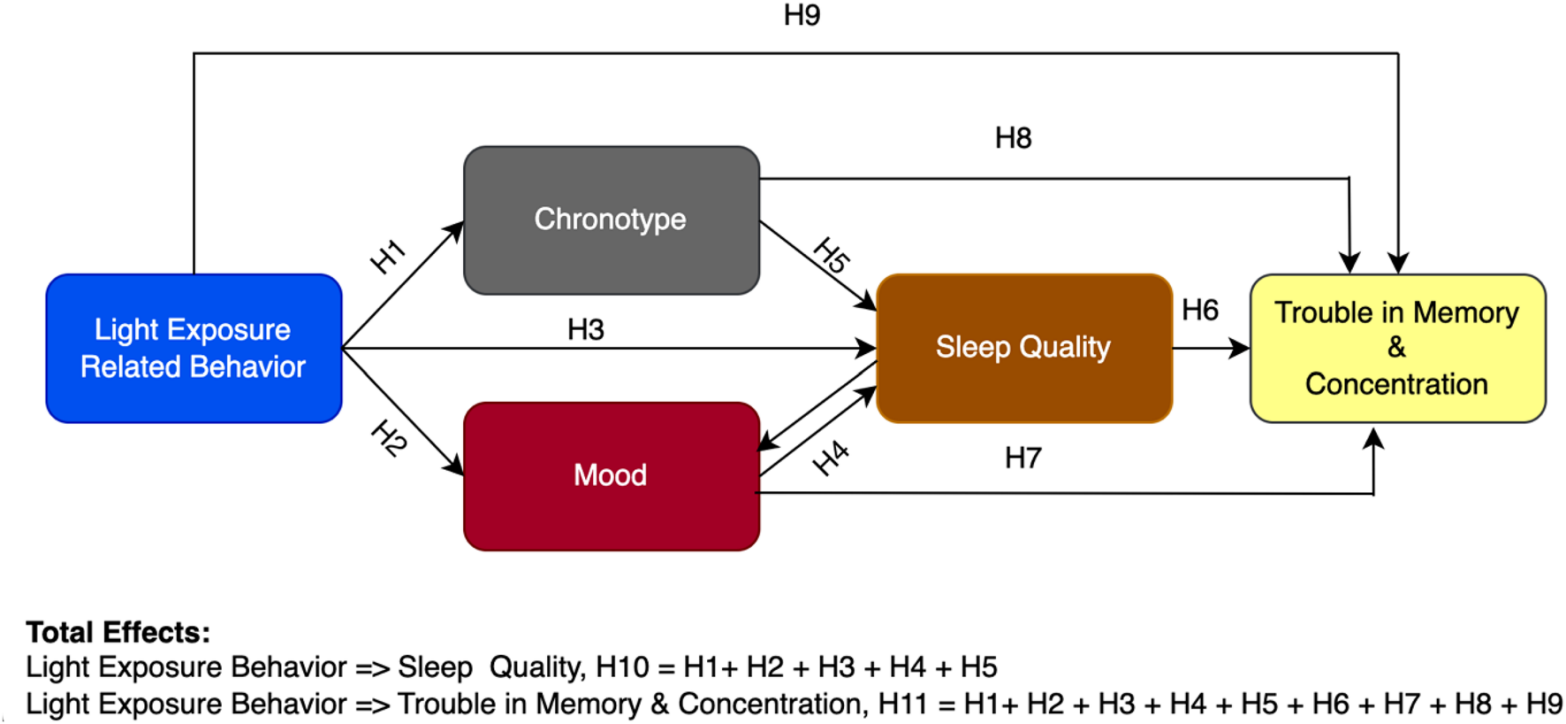
Theoretical framework of the fitted PLS-SEM model to predict chronotype, sleep quality, mood, memory and concentration using LEBA categories as predictors.

We posed the following questions: What are the influences of LEBA categories on (a) chronotype, (b) mood, (c) sleep quality, and (d) memory and concentration? To answer these questions, we proposed a theoretical framework (Fig. 1) based on the literature reviewed. We used the partial least squares structural equation modeling (PLS-SEM)—most suited to formulate such a predictive model^57,58^. Predicting relationships using PLS-SEM is a two-step process. First, a measurement model is used to assess the reliability and validity of the latent variables used in the model. Second, a structural model is used to investigate the predicted relationships of the latent structures. In the structural model, (i) the direct effects (DE): influences unmediated by any other constructs in the model, (ii) indirect effects (IE): influences mediated by at least one intervening construct in the model and (iii) total effects (TE): sums of direct and indirect effects of a given construct can be estimated^59^.

We predicted that five types of LEBA behavior categories would directly influence chronotype (H1), mood (H2), and sleep quality (H3). We also predicted a bidirectional relationship between mood and sleep quality (H4). Additionally, we predicted that chronotype (H5) would influence sleep quality. Sleep quality (H6), mood (H7), and chronotype (H8) would affect memory and concentration. LEBA categories would directly influence memory and concentration (H9). Lastly, we predicted that LEBA categories would exhibit a significant total effect on sleep quality (H10), memory, and concentration (H11).

## Methods

### Participants

We conducted a large-scale online survey on Malaysian residents. The exclusion-inclusion criteria for respondents to be included in this study were: (1) any Malaysian resident aged > 18 and able to read and write English (2) no physiological and psychological disorder (self-reported). Three hundred and sixty-six adults completed the survey. The completion rate of our survey was 87% (45 participants' data was excluded due to incompleteness). We further excluded 19 participants based on our exclusion-inclusion criteria. Thus, we used data from 301 participants for further processing.

A priori power analysis was conducted to determine the sample size adequacy with G^*^Power 3.0^60^. To achieve an effect size of 0.15^61^ and 80% statistical power and $\alpha$ = 0.05, for a multiple liner regression with 13 predictors, a total sample size of 131 individuals was needed. Further, the maximum number of items per factor in our model was six. In the PLS-SEM-based analysis, to detect a minimum ${R}^{2}$ value of 0.10 for a factor with six items with 80% statistical power and* α* = 0.05, at least 130 participants are required^62^. Our sample size exceeded these recommendations.

### Measures

#### Light exposure behavior assessment

Light exposure-related behaviors were measured using the short form of the Light Exposure Behavior Assessment^56^. The short form contains five factors with 18 items. Light Exposure Behavior Assessment (LEBA) measures the propensity of different light exposure-related behaviors in the last month retrospectively using a five-point Likert-type response scale (1 = never; 2 = rarely; 3 = sometimes; 4 = often; 5 = always).

#### Positive and negative affect schedule

The positive and negative affect schedule (PANAS)^63^ was used to measure positive (PA) and negative affect (NA) with two 10-item mood scales. Participants retrospectively rated their positive and negative affect based on the last month using a five-point Likert-type response scale (1 = very slightly/not at all; 2 = a little; 3 = moderately; 4 = quite a bit; 5 = extremely).

#### Memory and concentration

We developed two single global items with four-point Likert-type response options investigating trouble in memory and concentration. These single global items asked the participants about the propensity of their memory and concentration difficulty in the last month (0 = Absent; 1 = Slight; 2 = Moderate; 3 = Severe).

#### Pittsburgh sleep quality index

We used the Pittsburgh Sleep Quality Index (PSQI)^64^ to measure the participants' sleep quality. PSQI measures seven domains of sleep to differentiate “poor” from “good” sleep. Participants responded to the PSQI using Likert-type response options ranging from 0 to 3, whereby 3 reflects the negative extreme on the Likert Scale. A sum of scores ≥ 5 indicates poor sleep quality. The latent structure of PSQI was reported to vary from one factor to three factors^64,65^. Dunleavy et al.^66^, in their study, recommended using a two-factor model: perceived sleep quality (PSQ) and sleep efficiency (SE) while measuring the sleep quality among Singapore citizens. In this study, we followed their recommended structure. A higher score in the PSQ factor would indicate decreased perceived sleep quality. In contrast, a higher score in the SE factor would indicate higher sleep efficiency.

#### Morningness-eveningness questionnaire

Chronotype was measured using Morningness-Eveningness questionnaire (MEQ)^67^. MEQ consists of 19 questions, and the scores range from 16 to 86. A higher score indicates a higher morning propensity. Caci et al.^68^ reported a four-factor structure of MEQ: peak time (PT), morning affect (MA), retiring time (RT) and rising time (RI) in a student sample (*N* = 456). Items in PT assess the body’s peak time for different activities. MA assesses our bodily responses in the morning. RT assesses the time when our body starts to prepare for sleeping. Lastly, RI investigates the time when our body prepares for waking up.

### Procedure

The project was approved by Monash University Human Research Ethics Committee (Project ID: 14,786). The research was performed in accordance with the relevant guidelines/regulations of the Declaration of Helsinki. Informed consent was obtained from all participants before data collection. This was a cross-sectional, fully anonymous online survey. Participants were invited via email and social media (i.e., LinkedIn, Twitter, and Facebook) with the attachment of an Explanatory Statement in which we mentioned that their participation would be voluntary and that they could withdraw from participation at any time without being penalized. If the participants expressed happiness with the statement, a survey link was sent to them. At the beginning of the survey, their consent was recorded digitally. The survey took 15–20 min, for which they were not compensated. The survey was completed between April and November, 2022.

### Analytic strategies

We used R (4.1.2v)^69^ and several statistical packages, including esemComp^70^, “SEMinR^71^” and tabledown^72^ for our analysis.

#### Structural validity of the scales

We gathered structural validity evidence of LEBA, PSQI, MEQ and PANAS scales in our sample using the exploratory structural equation modeling (ESEM)^73^. ESEM intricates the computational advantages of exploratory and confirmatory factor analysis by allowing the items to cross-load to represent the data more realistically and offering fit indices to assess the model fit. To assess the model fit, we followed the guidelines of Hu and Bentler^74^: comparative fit index (CFI) and the Tucker Lewis index (TLI): acceptable fit $\ge 0$.90, good fit $\ge 0$.95; the root mean square error of approximation (RMSEA): acceptable fit < 0.08, good fit < 0.06; and the standardized root mean square (SRMR): acceptable fit < 0.10, good fit < 0.08.

#### Partial least squares structural equation modeling

##### Measurement model assessment

First, we assessed the quality of the measurement model. We excluded items with factor loading < 0.40 to increase the robustness of the measurement model^71^. Second, we estimated the internal consistency reliability estimates of each construct. We reported both the lower bound estimate of reliability- Cronbach’s $\alpha$ coefficient and the upper bound estimate of reliability-construct reliability (CR). Both Cronbach’s $\alpha$ and *CR* coefficient values range between 0 and 1, where higher values represent better reliability. As a general guideline, Cronbach’s $\alpha$ above 0.70 is considered satisfactory^75,76^ and a value above 0.50 is considered acceptable^77^. *CR* coefficient value of 0.60 and above indicates a satisfactory reliability^71^.

Third, we assessed the convergent and discriminant validity of the measurement model. For *convergent validity*, we used each construct's average variance extracted (AVE) value. *AVE* ≥ 0.50 or *AVE* < 0.50 with a *CR* > 0.60 and *AVE* < *CR* indicate an acceptable convergent validity^78^. For *discriminant validity,* we compared the square root of the AVE of a construct with its corresponding correlation with other constructs^78^. The square root of the AVEs of each construct should be higher than its correlation with other constructs. We have also reported the bootstrapped heterotrait-monotrait ratio (*HTMT*) of correlations of the construct as additional proof of discriminant validity. For conceptually similar constructs, the *HTMT* value should be < 0.90; for constructs that are conceptually distinct, the *HTMT* value should be < 0.80^79^.

##### Structural model assessment

**Figure 2:**
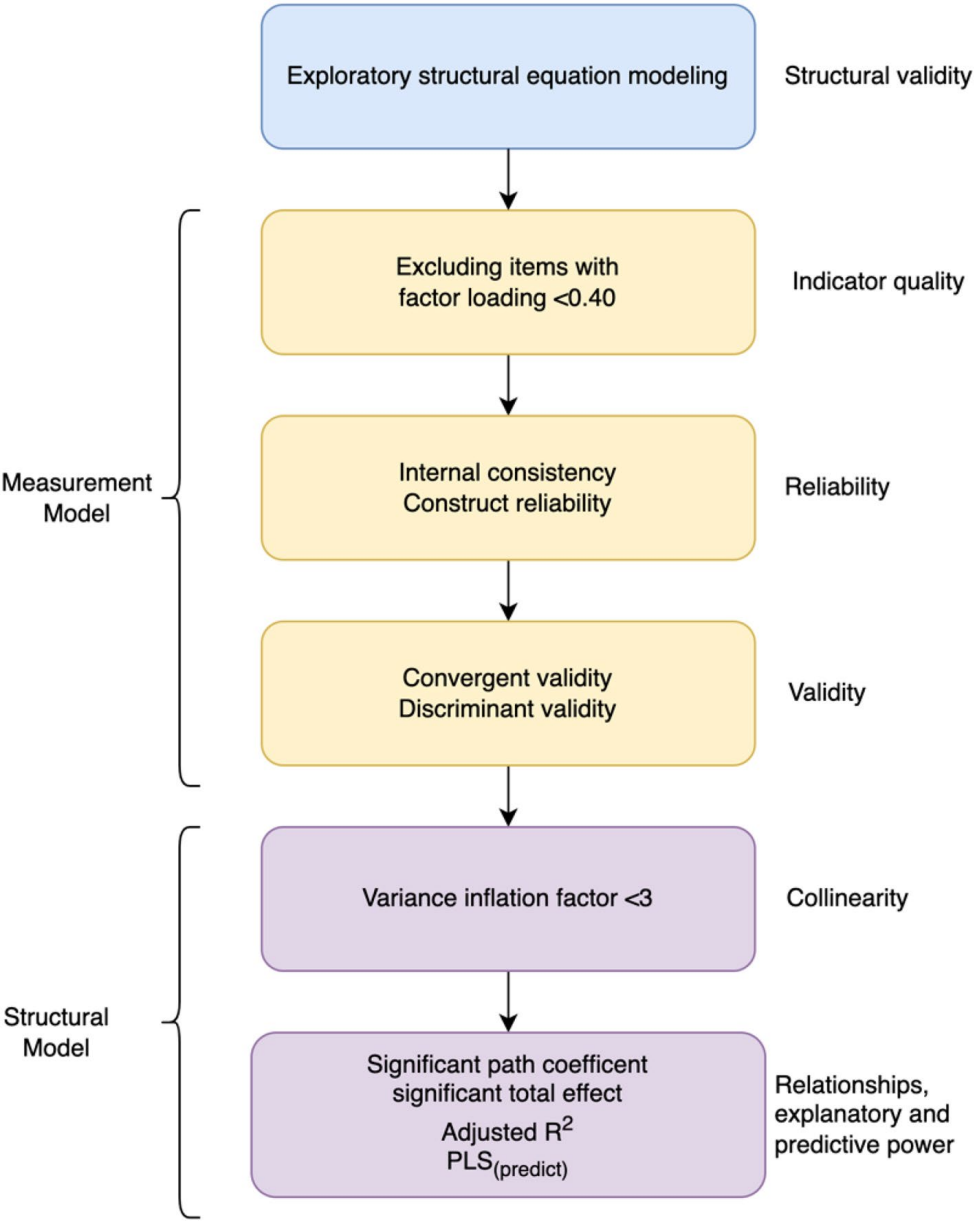
Analyses steps used in the study.

First, we assessed the collinearity of the constructs in our structural model by calculating variance inflation factor (*VIF*) values. *VIF* > 3 indicates probable collinearity issues^79^. Next, we estimated the direct effects (*DE*) and total effects (*TE*) of the structural model using a bootstrapping approach with 10,000 sub-samples and reported the significant total effects (*t* > 1.96) observed in our model. Lastly, we reported the adjusted ${R}^{2}$ as a measure of the explanatory power. For assessing the explanatory power, we followed the guidelines of Falk and Miller^80^: ${R}^{2}$ values $\ge$ 0.10 indicates adequate explanatory power. Further, we have categorized the ${R}^{2}$ values following the guidelines of Cohen^61^: 0.02 (weak), 0.13 (moderate), and 0.26 (substantial). For predictive relevance, we assessed the fitted model’s predictive power by K-fold cross-validation using the $PL{S}_{predict}$ function from the “SEMinR” package^71^. $PL{S}_{predict}$ provides the root-mean-square error (RMSE) and respective linear-regression model benchmarks (LM) for all indicators. We assessed the model’s predictive power by following the guideline of Hair^71^: (i) high predictive power: all indicators in the fitted PLS-SEM model have lower RMSE values compared to the LM (ii) medium predictive power: the majority(≥ 50%) of the indicators have lower RMSE values than LM (iii) low predictive power: less than 50% of the indicator have lower RMSE value than LM (iv) no predictive power: no indicator has lower RMSE value than LM model. Figure 2 depicts the analysis steps we followed.

### Ethics approval

The project received ethics clearance from Monash University Human Research Ethics Committee (Project ID: 14,786). Informed consent was obtained from all participants. Participants were assured about their voluntary participation and the right to withdraw at any time.

## Results

### Demographic and descriptive statistics

**Figure 3:**
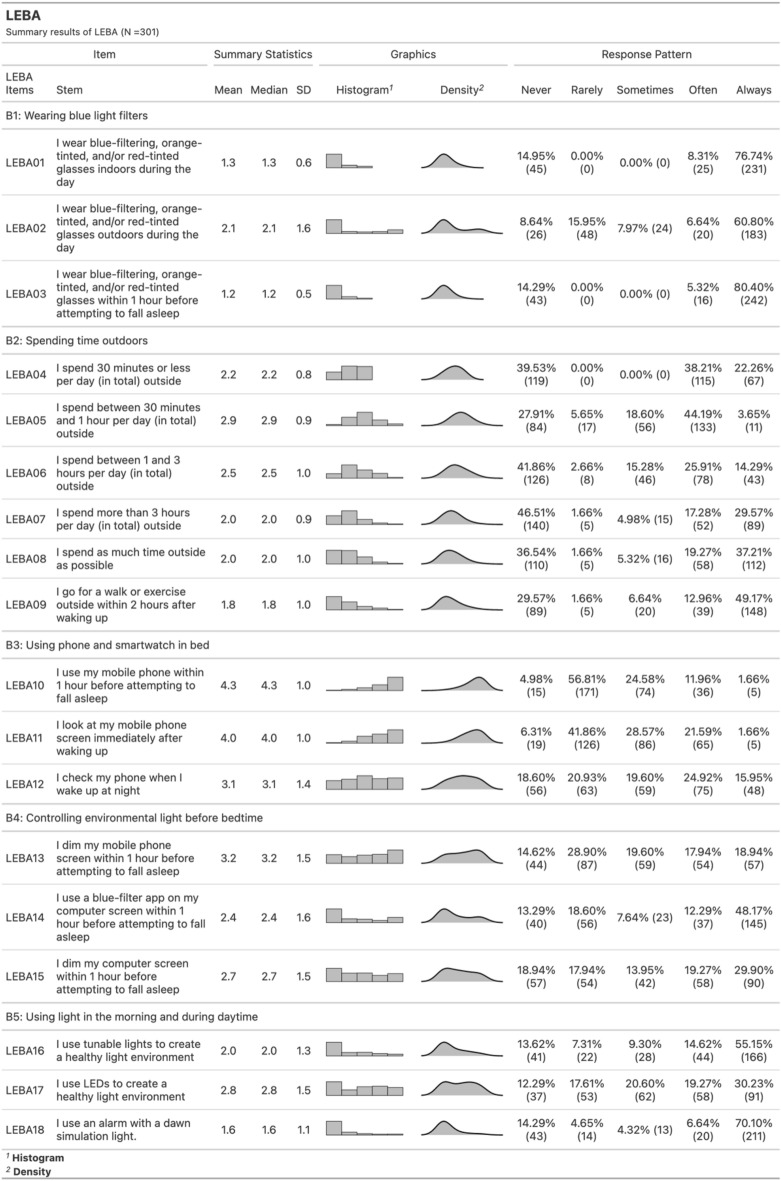
Response distribution of Light Exposure Behavior Assessment.

Out of 301 participants (Age_Mean±SD_ = 28 ± 9), 218 (72.43%) were female, ranging in age from 18 to 59 years (Age_Mean±SD_ = 26.85 ± 8.07), and 83 (27.57%) were male with an age range between 18 to 74 years (Age_Mean±SD_ = 30.35 ± 12.14): 78.66% were unmarried. The majority of the participants were students (71.42%) and of intermediate chronotype (68%). Table 1 summarizes the participants' demographics and descriptive statistics of the measures. Figure 3. depicts the response distribution, mean score and SD for each item of LEBA.

**Table 1:** Demographics and descriptive statistics of the participants (*N* = 301).

Characteristic	Female, *N* = 218 Mean (SD) or N(%)	Male, *N* = 83 Mean (SD) or N(%)
Age	27 (8)	30 (12)
**Religion**		
Atheist	23 (11%)	7 (8.4%)
Buddhist	99 (45%)	35 (42%)
Christian	36 (17%)	13 (16%)
Hindu	21 (9.6%)	11 (13%)
Muslim	39 (18%)	17 (20%)
**Ethnicity**		
Malaysian Chinese	138 (63%)	46 (55%)
Malaysian Indian	19 (8.7%)	13 (16%)
Malaysian Malay	26 (12%)	7 (8.4%)
Others	35 (16%)	17 (20%)
**Marital status**		
Single	180 (83%)	56 (67%)
Married	37 (17%)	27 (33%)
Divorced	1 (0.5%)	0 (0%)
**Education**		
Doctor of philosophy (PhD)	43 (20%)	13 (16%)
Master’s degree	38 (17%)	22 (27%)
Postgrad diploma	1 (0.5%)	0 (0%)
Bachelor’s degree	129 (59%)	41 (49%)
Diploma	7 (3.2%)	7 (8.4%)
**Occupation**		
Student	165 (76%)	50 (60%)
Work	42 (19%)	31 (37%)
Neither	11 (5.0%)	2 (2.4%)
**Community stance**	7.07 (1.87)	7.00 (1.85)
**Sleep quality**		
Good sleep	69 (32%)	24 (29%)
Poor sleep	149 (68%)	59 (71%)
**Chronotype**		
Definite evening	8 (3.7%)	1 (1.2%)
Intermediate	144 (66%)	60 (72%)
Moderate evening	43 (20%)	13 (16%)
Moderate morning	23 (11%)	9 (11%)
Definite morning	0 (0%)	0 (0%)
**Time of participating in the survey**		
Morning (6AM-11:59AM)	24 (11%)	9 (11%)
Afternoon (12 PM-5PM)	101 (46%)	42 (51%)
Evening (5:01 PM- 8PM)	75 (34%)	22 (27%)
Night (8:01 PM-5:59PM)	18 (8%)	10 (12%)
**Descriptive Statistics of the Measures**		
Wearing blue light filter glasses indoors and outdoors (LEBA B1)	4.75 (5.32)	3.49 (4.56)
Spend time outdoors (LEBA B2)	3.96 (2.58)	4.20 (2.32)
Usage of mobile phones on the bed before sleeping (LEBA B3)	8.06 (3.86)	8.96 (3.56)
Controlling environmental light before bedtime (LEBA B4)	8.31 (3.49)	8.33 (3.60)
Use of electric light (LEBA B5)	6.48 (2.94)	6.28 (2.32)
Positive Affect	27.77 (8.67)	28.99 (8.01)
Negative Affect	23.27 (5.77)	22.78 (5.27)
Pittsburgh sleep quality index (PSQI)	5.70 (2.44)	6.34 (3.09)
Morningness-eveningness questionnaire (MEQ)	47.96 (8.62)	48.90 (7.50)
Trouble in memory	1.17 (0.93)	1.12 (0.85)
Trouble in concentration	1.54 (0.88)	1.42 (0.83)

### Structural validity

Table 2 presents the fit indices of the scales used in this study. Light Exposure Behavior Assessment (LEBA), Pittsburgh Sleep Quality Index (PSQI), Morningness*-*Eveningness Questionnaire (MEQ), and Positive and Negative Affect Scales (PANAS) exhibited acceptable to a good fit in terms of *CFI* and *TLI* (> 0.95 or 0.90), *RMSEA* (< 0.08 or 0.06), and *SRMR* (< 0.08).

**Table 2:** Structural validity of the scales used in the PLS-SEM model assessed using ESEM.

	*χ*^2^	Degrees of freedom, p	Comparative fit index (CFI)	Tucker Lewis index (TLI)	Root mean square error of approximation (RMSEA;90% CI)	Standardized root mean square residual (SRMR)
LEBA	128.99	73, *p* < 0.001	0.977	0.951	0.05(0.04–0.07)	0.04
PSQI	19.84	8, *p* = *0.011*	0.966	0.910	0.07(0.03–0.11)	0.07
MEQ	91.50	101, *p* < 0.001	0.970	0.949	0.04(0.03–0.06)	0.04
PANAS	293.76	151, *p* < 0.001	0.992	0.990	0.06(0.05–0.07)	0.06

### Measurement model

We excluded two items from LEBA (items 02 & 04) and four items from MEQ* (*items 06, 10, 16, 12) due to weak factor loadings (< 0.40; Supplementary Table S1). The results of the refitted measurement model assessment are presented in Supplementary Table S2. The sleep efficiency (SE) factor of PSQI exhibited poor reliability in terms of Cronbach’s $\alpha$ ($\alpha$= 0.48) but had satisfactory construct reliability (*CR* = 0.79). All other factors exhibited acceptable to satisfactory internal consistency in terms of Cronbach’s $\alpha$ (0.51–0.94) and construct reliability (0.72–0.96). Concerning convergent validity, 8 out of 13 constructs had average variance extracted (*AVE*) > 0.50 (except LEBA B2, negative affect, perceived sleep quality, peak time and retiring). However, all 13 constructs had *CR* > 0.60 and *AVE* < *CR*. This indicated acceptable reliability and convergent validity of all constructs in the model.

To establish the discriminant validity, we calculated the square root of each construct’s *AVE* and compared them to their corresponding inter-construct correlation (Supplementary Table S3). All constructs’ square root of *AVE* were greater than their inter-construct correlation indicating satisfactory discriminant validity. Further evidence of the discriminant validity of the constructs was drawn by heterotrait-monotrait (*HTMT)* analysis. Supplementary Table S4 presents the HTMT values and indicates satisfactory discriminant validity (*HTMT* < 0.80) for all 13 constructs.

### Structural model

Variance inflation factors (*VIF*) for all constructs were < 3, indicating no possible collinearity. Table 3 depict significant (*t* > 1.96) direct effects and total effects observed in our model. All direct effects of the structural model are provided in Supplementary Table S5.

**Table 3:** Direct and total effects estimated in the PLS-SEM model (Only significant structural relationships are reported, *t* > 1.96).

Hypothesis	Path coefficients	Original Estimates	Bootstrap mean	Bootstrap SD	t	2.5% CI	97.5% CI	Results
Direct effects								
H1: Light exposure-related behaviors—> Chronotype								
H1	LEBA B1—> PT	− 0.25	− 0.22	0.08	− 2.91	− 0.36	− 0.01	Supported
	LEBA B2—> PT	0.20	0.19	0.07	3.03	0.06	0.31	
	LEBA B2—> RT	0.17	0.17	0.06	2.69	0.04	0.29	
	LEBA B2—> RI	0.14	0.13	0.06	2.22	0.01	0.25	
	LEBA B3—> PT	− 0.22	− 0.23	0.05	− 4.13	− 0.33	− 0.12	
	LEBA B3—> MA	− 0.12	− 0.12	0.06	− 2.09	− 0.23	0.01	
	LEBA B3—> RT	− 0.25	− 0.25	0.05	− 4.61	− 0.36	− 0.15	
	LEBA B3—> RI	− 0.23	− 0.24	0.06	− 3.96	− 0.35	− 0.12	
	LEBA B5—> PT	0.15	0.15	0.06	2.34	0.02	0.27	
	LEBA B5—> MA	0.14	0.14	0.07	2.02	0.00	0.27	
	LEBA B5—> RT	0.15	0.14	0.07	2.15	0.01	0.27	
H2: Light exposure-related behaviors—> Mood								
H2	LEBA B2—> PA	0.33	0.33	0.05	6.32	0.22	0.42	Supported
	LEBA B5—> NA	0.19	0.18	0.09	2.13	− 0.02	0.34	
H3: Light exposure-related behaviors—> sleep quality								
H3	LEBA B3—> PSQ	0.13	0.13	0.06	2.21	0.01	0.24	Supported
	LEBA B5—> PSQ	− 0.16	− 0.15	0.06	− 2.57	− 0.27	− 0.03	
H4: Mood < = > Sleep quality								
H4	PA—> PSQ	− 0.19	− 0.19	0.06	− 3.05	− 0.30	− 0.06	Supported
	PA—> SE	0.21	0.21	0.07	3.00	0.07	0.34	
	NA—> PSQ	0.28	0.29	0.06	4.97	0.18	0.40	
	PSQ—> PA	− 0.29	− 0.29	0.05	− 5.39	− 0.39	− 0.18	
	PSQ—> NA	0.37	0.38	0.05	7.17	0.27	0.48	
	SE—> PA	0.21	0.21	0.05	3.84	0.10	0.31	
H5: Chronotype—> Sleep Quality								
H5	MA—> PSQ	− 0.21	− 0.20	0.06	− 3.43	− 0.32	− 0.09	Supported
H6: Sleep quality—> memory and concentration								
H6	PSQ—> Memory	0.13	0.14	0.06	2.27	0.02	0.25	Supported
	PSQ—> Concentration	0.22	0.23	0.06	3.87	0.11	0.31	
H7: Mood—> Memory and Concentration								
H7	NA—> Memory	0.37	0.36	0.06	6.30	0.25	0.47	Supported
	NA—> Concentration	0.32	0.31	0.06	5.75	0.20	0.42	
H8: Chronotype—> Memory and Concentration								
	MA—> Memory	− 0.13	− 0.14	0.06	− 2.27	− 0.25	− 0.02	
	MA—> Concentration	− 0.11	− 0.11	0.06	− 2.05	− 0.22	0.00	
H9: Light exposure related behavior—> Memory and concentration								
H9	Details are provided Supplementary Table 5							Not Supported
Total Effects								
Hypothesis	Path coefficients	Original Est	Bootstrap Mean	Bootstrap SD	T Stat	2.5% CI	97.5% CI	Results
H10: LEBA to Sleep quality								
H10	LEBA B3—> PSQ	0.20	0.20	0.06	3.41	0.08	0.32	Supported
	LEBA B5—> PSQ	− 0.18	−0.18	0.07	− 2.53	− 0.31	− 0.03	
H11: LEBA to memory and concentration								
H11	LEBA B3—> Memory	0.20	0.19	0.06	3.17	0.06	0.31	Supported
	LEBA B3—> Concentration	0.23	0.23	0.06	3.82	0.11	0.34	

### Predicted relationships

Table 3 shows that, in line with our predictions, LEBA categories exhibited direct effects on chronotype *(H1)*, mood *(H2)* and sleep quality *(H3).* Among the four factors of chronotype, we observed a negative direct effect of wearing blue light filter glasses (LEBA B1) on peak time (PT; body’s peak time for different activities). Less use of blue light filter glasses indoors during the day and more within one hour before sleep (LEBA B1) predicted early peak time (PT; $\beta$= −0.25). Spending time outdoors (LEBA B2) exhibited a direct effect on positive affect ($\beta$= 0.33) and chronotype factors: peak time (PT; $\beta$= 0.20), retiring time (RT; the time when our body starts to prepare for sleeping; $\beta$= 0.17), and rising time (RI; the time when our body prepares for waking up; $\beta$= 0.14). In contrast, the usage of mobile phones on the bed before sleeping (LEBA B3) directly but negatively, influenced the four chronotype factors: PT ($\beta$= −0.22), morning affect (MA; $\beta$= −0.12), RT ($\beta$= −0.25) and RI ($\beta$= −0.23) and predicted decreased perceived sleep quality (PSQ; $\beta$= 0.13; a higher score indicated poor sleep quality). The increased use of electric light during daytime (LEBA B5) positively influenced chronotype factors: PT ($\beta$= 0.15), MA ($\beta$= 0.14) and RT ($\beta$= 0.15) and increased perceived sleep quality (PSQ; $\beta$= −0.16; a lower score indicated higher sleep quality). But this behavior dimension (LEBA B5) was associated with increased negative affect ($\beta$= 0.19). Results indicated a bidirectional relationship between mood and sleep quality. Positive and negative affect directly influenced sleep quality, and vise-versa *(H4).* Positive mood increased both the sleep efficiency (SE; $\beta$= 0.21) and PSQ ($\beta$= −0.19), whereas negative affect decreased PSQ ( $\beta$= 0.28). Again, better SE and PSQ predicted better PA ($\beta$= 0.21 and −0.29). Better PSQ was predicted less NA ($\beta$= 0.37).

Chronotype directly influenced sleep quality *(H5)*, where increased MA was observed to increase PSQ ($\beta$= −0.21). A negative influence of PSQ was observed on memory and concentration *(H6),* whereby poor PSQ was predicted to increase trouble in memory ($\beta$= 0.13) and concentration ($\beta$= 0.22). Increased negative affect predicted a deteriorated memory and concentration (*H7*; memory = 0.37; concentration = 0.32). We also observed direct effect of chronotype on trouble in memory and concentration *(H8)*. Increased morning affect was predicted to decrease trouble in memory and concentration ($\beta$= −0.13 and −0.11). However, no significant direct effect of light exposure-related behaviors *(H9)* was observed on trouble in memory and concentration.

We observed significant total effects of light exposure-related behaviors on sleep quality *(H10)*. The usage of mobile phones on the bed before sleeping (LEBA B3) predicted the decrease of PSQ ($\beta$= 0.20), whereas increased use of electric light during daytime (LEBA B5) increased PSQ ($\beta$= −0.18). Lastly, significant total effects of light exposure-related behaviors on memory and concentration were observed *(H11)*. The usage of mobile phones on the bed before sleeping (LEBA B3) predicted an increase of memory and concentration problem ($\beta$= 0.20 and $\beta$= 0.23, respectively).

### Explanatory and predictive power of the fitted model

Our fitted model exhibited substantial explanatory power (*R*^*2*^) for PSQ (26.70%), trouble in concentration (31.67%) and trouble in memory (27.32%). Moderate *R*^*2*^ was observed for PA (25.27%), NA (18.03%), PT (14.58%) and RT (13.32%). Our model exhibited weak *R*^*2*^ for MA (4%), RI (9%), and SE (4%). $PL{S}_{predict}$ function indicated our model had medium predictive power with 72.72% of the indicators having RMSE value lower than the LM benchmark.

## Discussion

This study investigated if light exposure behaviors predict chronotype, sleep quality, mood, memory, and concentration. Preliminary analyses revealed that light-exposure behaviors affected sleep quality, concentration, and memory. Results from LEBA indicated that participants generally used blue light filters less often, spent less time outdoors, and were highly engaged in mobile phones in bed before sleep. These behaviors could have contributed to poor sleep quality and trouble in memory and concentration. The results strengthened the need for a model to predict how light exposure behaviors explain human cognition and sleep quality.

The measurement models indicated acceptable reliability and validity of the scales we used to measure chronotype, sleep quality, and mood. Two factors: sleep efficiency (SE) and rising time (RI), had Cronbach’s *α* < 0.60 but exhibited satisfactory construct reliability (> 0.60). These two factors were composed of only two items each, which might have contributed to the low Cronbach’s alpha coefficient. Further, we only asked two separate questions to assess if the participants experienced any trouble with recalling memory and concentration. Using such global single items allowed us to reduce participants’ cognitive demands required to respond to the survey and increased the response rate^81^. Typically, single questions are found reliable with good predictive validity and allow the participants to consider the key features of the given construct^82–85^.

Results indicated that the structural model had satisfactory explanatory power (*R*^*2*^ > 0.10) for all factors except for morning affect (MA), rising time (RI), and sleep efficiency (SE). These three factors exhibited weak *R*^*2*^. One possible reason could be that they are influenced by other factors not included in the model, such as genetics, time of day, and work schedule. In any case, our models generally exhibited satisfactory predictive relevance, and most relationships confirmed our predictions.

*Wearing blue light filters* (LEBA B1) influenced peak time directly—a chronotype indicating the body’s peak time for different activities. Lower usage of blue light filters indoors during the day and higher usage at night, especially one hour before sleep, predicted a circadian phase advancement, meaning our body starts functioning earlier than the usual time (Direct effect, *DE* = −0.21). The results support previous studies that showed the blue light exposure during daytime and the absence of blue light at night was responsible for synchronizing our body clock with the natural light–dark cycle and preparing our body to rise early^86,87^. A group of photoreceptors in our eye—intrinsically photoreceptive retinal ganglion cells (ipRGCs) are sensitive to blue light^2,6^. These ipRGCs receive signals from the light and send them to the suprachiasmatic nucleus (SCN) region of our brain, the so-called master clock of our body clock, to align our inner rhythm with the astronomical cycle. Hence, deprivation of blue light during the daytime, especially in the morning, and exposure at night misguides our circadian rhythm. Figueiro et al.^88^ reported that blue-enriched light exposure throughout the day promotes better alignment of the circadian rhythm with the earth’s 24-h light–dark cycle. Figueiro and Rea^89^ observed a delay in nighttime melatonin onset due to blue-depleted daytime light exposure (from the awakening time until approximately 15:00), causing a circadian phase delay.

*Spending time outdoors *(LEBA B2) predicted an improved mood in our participants by increasing their positive affect. Previous studies also reported similar results^8,90^. An et al.^90^ observed reduced depressive mood in workers when more sunlight was available in their workplace. Figueiro et al.^8^ found fewer depressive symptoms for light exposures with high circadian efficiency—an ability to entrain our body clock like the sunlight. We observed a positive direct effect of spending time outdoors on chronotype. It indicates a potential relationship between exposure to outdoor light and phase advancement in our circadian rhythm (see also^8,91^). After analyzing a bio-bank of 400,000 UK participants, Burns et al.^91^ reported that time spent in outdoor light promoted phase advancement. Also, there could be a possibility that people who have early chronotypes might have the advantage of spending more time outdoors than those with late chronotypes. The results suggest that sleep and mood-related problems could be rooted in people’s light exposure-related behaviors.

*Increased use of smart gadgets (mobile phones)* in bed before night sleep (LEBA B3) predicted the phase delay and reduced sleep quality. This exposure-related behavior also harmed memory performance and concentration. Past research revealed adverse effects of using smart devices in bed on sleep quality^92–94^. The self-luminous display of smart gadgets often emits blue lights, exposure to which at night is directly associated with reduced cognitive functioning, circadian phase shift, and reduced sleep quality^95–99^.

Results indicated that the increased *use of electric light (tunable, LED, or dawn-simulating light) in the morning and daytime* (LEBA B5) increased sleep quality and predicated a circadian phase advancement. Figueiro et al.^8^ found similar results, whereby increased circadian daytime light exposure improved sleep quality among office workers. Several studies independently demonstrated that inadequate daytime light exposure caused increased melatonin suppression at night, resulting in a circadian phase delay, more nighttime awakening, sleep deprivation, and poor sleep quality^100–102^. Studies based on real-world settings such as offices and schools also indicated that increased electric light exposure improved sleep quality^9,103,104^. However, increased use of these electric lights in the morning also predicted increased negative affect. The use of electric lights in the morning and during the day could be associated with the inaccessibility of sunlight, which might contribute to increased negative affect^105^.

Unexpectedly, we did not observe any influence of the factor—*Controlling the light environment before bedtime* (LEBA B4) on sleep, emotion, and cognition. The participants reported if they controlled the light emitted from their devices before bedtime, such as if they used blue light filter applications or dimmed the monitor one hour before sleep. But, recent recommendations indicated that investigations related to light in a sleep environment should consider a time span of three hours prior to sleep^55^, which could be a contributing factor to such findings. Additionally, the effects of light before bedtime could also depend on light exposure history and characteristics of surrounding light^106^, which were not accounted for in our model.

Results indicated that increased *morning affect (H8),* a factor of chronotype, predicted less trouble in memory and concentration*.* Although the circadian phase advancement enhanced memory and concentration, the relationship seemed more complex because other factors might influence this relationship. For example, early chronotypes may be less susceptible to social jetlag (misaligned sleep–wake pattern with work schedule); hence, people might subsequently experience fewer issues with memory and concentration than other chronotypes^107,108^.

We like to mention several limitations of this study. First, we fitted the PLS-SEM-based model on a female-dominated sample that hinders the generalizability of the results. Future studies should recruit a gender-balanced sample with higher representativeness of the multi-ethnic Malaysian population and fit the model to balanced subgroups such as ethnicity or age. Second, morning affect (MA), rising behaviors (RI), and sleep efficiency (SE) exhibited weak *R*^2^ in our fitted model. Further research with larger samples and more comprehensive measures for additional variables might be necessary to improve the explanatory power of the fitted model. Third, the importance of considering the time of the day when accounting for the effects of light exposure cannot be overstated. However, we should note that most of the behavioral dimensions of LEBA do not objectively address the time of the day. To improve the accuracy and reliability of future studies, we recommend that researchers consider the role of time of the day as a variable and develop a model that incorporates it. Fourth, there was an underrepresentation of elderly participants (> 65 years of age) in this study. Participants' age is a critical factor that can significantly influence light exposure-related behavior, which raises concerns about the generalizability of the study's findings to the older population.

## Conclusion

This research investigated whether light exposure-related behaviors could predict chronotype, sleep quality, mood, memory, and concentration. Our goal was to devise a healthy light diet. We first developed a conceptual framework and then applied a partial least square structural equation modeling to data gathered from 301 Malaysian adults. All the constructs used in the model exhibited acceptable reliability and validity. Results indicated that the less usage of wearable blue filters outdoors during the day and more within one hour before sleep predicted a circadian phase advancement. Also, spending time outdoors promotes mood and circadian phase advancement. However, using gadgets (mobile phones) in bed before sleeping negatively affected mood, sleep quality, memory, and concentration. The former also predicted a circadian phase delay. Using electric light (tunable, LED, or dawn-simulating light) in the morning and during the daytime promotes circadian phase advancement and enhances sleep quality. Generally, these findings would help develop a healthy light diet to facilitate health and wellness.

## Supplementary Information

Supplementary Tables.

## Data Availability

The datasets generated and/analyzed during the current study are available in the GitHub repository, https://github.com/ILLMMU/Study2.
