# Supplementary material for: Light exposure behaviors predict mood, memory and sleep quality
Source: Sci Rep. 2023 Aug 1;13:12425. doi: 10.1038/s41598-023-39636-y (PMC10394000; doi:10.1038/s41598-023-39636-y)
Supplement: Supplementary file 1 — Supplementary Tables. [file 41598_2023_39636_MOESM1_ESM.docx]

**Supplementary Materials**

**Supplementary Table S1:** Results of measurement model assessment

| Constructs | Factor Loading | Cronbach’s alpha | Construct  Reliability | Average Variance  Explained |
| --- | --- | --- | --- | --- |
| **LEBA B1** |  | 0.65 | 0.78 | 0.66 |
| item1 | 0.92 |  |  |  |
| item2 | 0.28 |  |  |  |
| item3 | 0.92 |  |  |  |
| **LEBA B2** |  |  |  |  |
| Item4 | 0.31 | 0.69 | 0.78 | 0.38 |
| item5 | 0.47 |  |  |  |
| item6 | 0.72 |  |  |  |
| item7 | 0.62 |  |  |  |
| item8 | 0.68 |  |  |  |
| item9 | 0.78 |  |  |  |
| **LEBA B3** |  | 0.71 | 0.84 | 0.64 |
| item10 | 0.85 |  |  |  |
| item11 | 0.86 |  |  |  |
| item12 | 0.68 |  |  |  |
| **LEBA B4** |  | 0.67 | 0.82 | 0.60 |
| item13 | 0.75 |  |  |  |
| item14 | 0.69 |  |  |  |
| item15 | 0.87 |  |  |  |
| **LEBA B5** |  | 0.51 | 0.74 | 0.50 |
| item16 | 0.76 |  |  |  |
| item17 | 0.54 |  |  |  |
| item18 | 0.74 |  |  |  |
| **Single Item Measures** |  |  |  |  |
| Trouble in Concentration | 1.00 | 1.00 | 1.00 | 1.00 |
| Trouble in Memory | 1.00 | 1.00 | 1.00 | 1.00 |
| **Perceived Sleep Quality (PSQ)** |  | 0.60 | 0.73 | 0.36 |
| Component 1 | 0.72 |  |  |  |
| Component 2 | 0.44 |  |  |  |
| Component 5 | 0.51 |  |  |  |
| Component 6 | 0.43 |  |  |  |
| Component 7 | 0.81 |  |  |  |
| **Sleep Efficiency (SE)** |  | 0.48 | 0.79 | 0.66 |
| Component 3 | 0.86 |  |  |  |
| Component 4 | 0.75 |  |  |  |
| **MEQ Peak Time (PT)** |  | 0.71 | 0.79 | 0.39 |
| Item 11 | 0.53 |  |  |  |
| Item 1 | 0.75 |  |  |  |
| Item 18 | 0.58 |  |  |  |
| Item 17 | 0.50 |  |  |  |
| Item 09 | 0.79 |  |  |  |
| Item 15 | 0.55 |  |  |  |
| **MEQ Morning Affect (MA)** |  | 0.53 | 0.70 | 0.48 |
| Item 07 | 0.85 |  |  |  |
| Item 04 | 0.79 |  |  |  |
| Item 05 | 0.73 |  |  |  |
| Item 06 | -0.15 |  |  |  |
| **MEQ Retiring (RT)** |  | 0.42 | 0.61 | 0.29 |
| Item19 | 0.75 |  |  |  |
| Item8 | 0.58 |  |  |  |
| Item 2 | 0.78 |  |  |  |
| Item 10 | 0.38 |  |  |  |
| Item 14 | 0.54 |  |  |  |
| Item 16 | -0.26 |  |  |  |
| Item 12 | 0.06 |  |  |  |
| **MEQ Rising (RI**) |  | 0.51 | 0.80 | 0.67 |
| Item 3 | 0.85 |  |  |  |
| Item 13 | 0.78 |  |  |  |
| **Positive Affect (PA)** |  | 0.92 | 0.93 | 0.57 |
| Interested | 0.74 |  |  |  |
| Excited | 0.72 |  |  |  |
| Strong | 0.84 |  |  |  |
| Enthusiastic | 0.81 |  |  |  |
| Proud | 0.71 |  |  |  |
| Alert | 0.63 |  |  |  |
| Inspired | 0.80 |  |  |  |
| Determined | 0.77 |  |  |  |
| Attentive | 0.72 |  |  |  |
| Active | 0.82 |  |  |  |
| **Negative Affect (NA)** |  | 0.86 | 0.89 | 0.45 |
| Distressed | 0.67 |  |  |  |
| Upset | 0.72 |  |  |  |
| Guilty | 0.64 |  |  |  |
| Scared | 0.74 |  |  |  |
| Hostile | 0.46 |  |  |  |
| Irritable | 0.68 |  |  |  |
| Ashamed | 0.65 |  |  |  |
| Nervous | 0.73 |  |  |  |
| Jittery | 0.58 |  |  |  |
| Afraid | 0.78 |  |  |  |

**Supplementary Table S2:** Results of measurement model assessment after discarding two item from Light Exposure Behavior Assessment (LEBA; item 02 & 04) and four items from Morningness-Eveningness Questionnaire (MEQ; items 06, 10,16,12).

| Constructs | Factor Loading | Cronbach’s alpha | Construct  Reliability (CR) | Average Variance  Explained (AVE) |
| --- | --- | --- | --- | --- |
| **LEBA B1** |  | 0.72 | 0.68 | 0.66 |
| item1 | 0.95 |  |  |  |
| item3 | 0.42 |  |  |  |
| **LEBA B2** |  | 0.71 | 0.80 | 0.45 |
| item5 | 0.46 |  |  |  |
| item6 | 0.73 |  |  |  |
| item7 | 0.62 |  |  |  |
| item8 | 0.69 |  |  |  |
| item9 | 0.79 |  |  |  |
| **LEBA B3** |  | 0.71 | 0.84 | 0.64 |
| item10 | 0.84 |  |  |  |
| item11 | 0.86 |  |  |  |
| item12 | 0.68 |  |  |  |
| **LEBA B4** |  | 0.67 | 0.82 | 0.60 |
| item13 | 0.73 |  |  |  |
| item14 | 0.69 |  |  |  |
| item15 | 0.89 |  |  |  |
| **LEBA B5** |  | 0.51 | 0.74 | 0.50 |
| item16 | 0.76 |  |  |  |
| item17 | 0.55 |  |  |  |
| item18 | 0.78 |  |  |  |
| **Single Item Measures** | |  |  |  |
| Trouble in Memory | 1.00 | 1.00 | 1.00 | 1.00 |
| Trouble in Concentration | 1.00 | 1.00 | 1.00 | 1.00 |
| **Perceived Sleep Quality (PSQ)** |  | 0.60 | 0.73 | 0.36 |
| Component 1 | 0.72 |  |  |  |
| Component 2 | 0.44 |  |  |  |
| Component 5 | 0.51 |  |  |  |
| Component 6 | 0.42 |  |  |  |
| Component 7 | 0.81 |  |  |  |
| **Sleep Efficiency (SE)** |  | 0.48 | 0.79 | 0.66 |
| Component 3 | 0.86 |  |  |  |
| Component 4 | 0.75 |  |  |  |
| **MEQ Peak Time (PT)** |  | 0.71 | 0.79 | 0.39 |
| Item 11 | 0.53 |  |  |  |
| Item 1 | 0.75 |  |  |  |
| Item 18 | 0.58 |  |  |  |
| Item 17 | 0.50 |  |  |  |
| Item 09 | 0.79 |  |  |  |
| Item 15 | 0.55 |  |  |  |
| **MEQ Morning Affect (MA)** |  | 0.72 | 0.84 | 0.64 |
| Item 07 | 0.87 |  |  |  |
| Item 04 | 0.80 |  |  |  |
| Item 05 | 0.73 |  |  |  |
| **MEQ Retiring (RT)** |  | 0.60 | 0.77 | 0.46 |
| Item19 | 0.76 |  |  |  |
| Item8 | 0.61 |  |  |  |
| Item 2 | 0.78 |  |  |  |
| Item 14 | 0.53 |  |  |  |
| **MEQ Rising (RI**) |  | 0.51 | 0.80 | 0.67 |
| Item 3 | 0.85 |  |  |  |
| Item 13 | 0.78 |  |  |  |
| **Positive Affect (PA)** |  | 0.92 | 0.93 | 0.57 |
| Interested | 0.74 |  |  |  |
| Excited | 0.72 |  |  |  |
| Strong | 0.84 |  |  |  |
| Enthusiastic | 0.81 |  |  |  |
| Proud | 0.71 |  |  |  |
| Alert | 0.63 |  |  |  |
| Inspired | 0.80 |  |  |  |
| Determined | 0.77 |  |  |  |
| Attentive | 0.72 |  |  |  |
| Active | 0.82 |  |  |  |
| **Negative Affect (NA)** |  | 0.86 | 0.89 | 0.45 |
| Distressed | 0.67 |  |  |  |
| Upset | 0.72 |  |  |  |
| Guilty | 0.64 |  |  |  |
| Scared | 0.74 |  |  |  |
| Hostile | 0.46 |  |  |  |
| Irritable | 0.68 |  |  |  |
| Ashamed | 0.65 |  |  |  |
| Nervous | 0.73 |  |  |  |
| Jittery | 0.58 |  |  |  |
| Afraid | 0.78 |  |  |  |

**Supplementary Table S3:** Discriminant validity assessment using the Fornell and Larcker Criterion. The bold numbers listed diagonally are the square root of the AVE of the constructs. The off-diagonals are the inter-correlations of the constructs for discriminant validity. The diagonal values should be larger than the values of the off-diagonals.

| Constructs | LEBA B1 | LEBA B2 | LEBA B3 | LEBA B4 | LEBA B5 | PT^1^ | MA^2^ | RT^3^ | RI^4^ | PA^5^ | NA^6^ | PSQ^7^ | SE^8^ | Memory | Concentration |
| --- | --- | --- | --- | --- | --- | --- | --- | --- | --- | --- | --- | --- | --- | --- | --- |
| LEBA B1 | **0.74** |  |  |  |  |  |  |  |  |  |  |  |  |  |  |
| LEBA B2 | 0.24 | **0.67** |  |  |  |  |  |  |  |  |  |  |  |  |  |
| LEBA B3 | -0.10 | -0.21 | **0.80** |  |  |  |  |  |  |  |  |  |  |  |  |
| LEBA B4 | 0.08 | 0.12 | 0.02 | **0.77** |  |  |  |  |  |  |  |  |  |  |  |
| LEBA B5 | 0.23 | 0.22 | -0.17 | 0.29 | **0.71** |  |  |  |  |  |  |  |  |  |  |
| PT | -0.15 | 0.21 | -0.26 | 0.00 | 0.16 | **0.63** |  |  |  |  |  |  |  |  |  |
| MA | -0.01 | 0.12 | -0.15 | 0.06 | 0.16 | 0.41 | **0.80** |  |  |  |  |  |  |  |  |
| RT | -0.02 | 0.21 | -0.30 | -0.09 | 0.16 | 0.64 | 0.38 | **0.68** |  |  |  |  |  |  |  |
| RI | 0.10 | 0.20 | -0.28 | -0.01 | 0.15 | 0.34 | 0.20 | 0.34 | **0.82** |  |  |  |  |  |  |
| PA | 0.08 | 0.35 | -0.11 | 0.02 | 0.21 | 0.33 | 0.31 | 0.28 | 0.18 | **0.76** |  |  |  |  |  |
| NA | 0.17 | 0.02 | 0.13 | 0.05 | 0.13 | -0.17 | -0.20 | -0.08 | -0.05 | -0.19 | **0.67** |  |  |  |  |
| PSQ | 0.02 | -0.06 | 0.23 | 0.02 | -0.18 | -0.25 | -0.35 | -0.19 | -0.12 | -0.33 | 0.37 | **0.60** |  |  |  |
| SE | 0.00 | 0.01 | -0.06 | -0.03 | 0.02 | 0.11 | 0.18 | 0.11 | 0.11 | 0.22 | -0.08 | -0.04 | **0.81** |  |  |
| Memory | 0.06 | -0.09 | 0.20 | 0.11 | 0.08 | -0.22 | -0.28 | -0.23 | -0.10 | -0.16 | 0.46 | 0.32 | -0.11 | **1.00** |  |
| Concentration | 0.01 | -0.05 | 0.23 | 0.06 | -0.04 | -0.26 | 0.46 | 0.43 | -0.15 | -0.26 | -0.31 | -0.16 | -0.17 | 0.52 | **1.00** |

^1: Peak time; 2: Morning affect; 3: RT: Retiring Time; 4: RI: Rising Time; 5: Positive affect; 6: Negative affect; 7: PSQ: Perceived sleep quality; 8: SE: Sleep efficiency^

**Supplementary Table S4:** Discriminant validity assessment using the HTMT. HTMT values indicate satisfactory discriminant validity. All HTMT values were<0.80.

| Constructs | LEBA B1 | LEBA B2 | LEBA B3 | LEBA B4 | LEBA B5 | PT^1^ | MA^2^ | RT^3^ | RI^4^ | PA^5^ | NA^6^ | PSQ^7^ | SE^8^ | Memory |
| --- | --- | --- | --- | --- | --- | --- | --- | --- | --- | --- | --- | --- | --- | --- |
| LEBA B2 | 0.52 |  |  |  |  |  |  |  |  |  |  |  |  |  |
| LEBA B3 | 0.36 | 0.26 |  |  |  |  |  |  |  |  |  |  |  |  |
| LEBA B4 | 0.31 | 0.23 | 0.09 |  |  |  |  |  |  |  |  |  |  |  |
| LEBA B5 | 0.55 | 0.40 | 0.28 | 0.52 |  |  |  |  |  |  |  |  |  |  |
| PT | 0.32 | 0.25 | 0.34 | 0.15 | 0.29 |  |  |  |  |  |  |  |  |  |
| MA | 0.22 | 0.15 | 0.20 | 0.08 | 0.27 | 0.52 |  |  |  |  |  |  |  |  |
| RT | 0.21 | 0.27 | 0.46 | 0.14 | 0.30 | 0.74 | 0.54 |  |  |  |  |  |  |  |
| RI | 0.24 | 0.26 | 0.44 | 0.14 | 0.28 | 0.52 | 0.33 | 0.57 |  |  |  |  |  |  |
| PA | 0.21 | 0.41 | 0.15 | 0.09 | 0.31 | 0.41 | 0.36 | 0.36 | 0.27 |  |  |  |  |  |
| NA | 0.33 | 0.16 | 0.21 | 0.11 | 0.29 | 0.26 | 0.25 | 0.17 | 0.15 | 0.25 |  |  |  |  |
| PSQ | 0.33 | 0.28 | 0.38 | 0.14 | 0.34 | 0.34 | 0.43 | 0.34 | 0.34 | 0.35 | 0.49 |  |  |  |
| SE | 0.20 | 0.06 | 0.17 | 0.17 | 0.13 | 0.21 | 0.31 | 0.25 | 0.22 | 0.32 | 0.13 | 0.23 |  |  |
| Memory | 0.16 | 0.12 | 0.24 | 0.13 | 0.10 | 0.26 | 0.32 | 0.26 | 0.14 | 0.16 | 0.49 | 0.35 | 0.16 |  |
| Concentration | 0.03 | 0.10 | 0.28 | 0.06 | 0.14 | 0.29 | 0.35 | 0.20 | 0.23 | 0.27 | 0.49 | 0.45 | 0.21 | 0.52 |

^1:PT- Peak time; 2: MA-Morning affect; 3: RT-Retiring Time; 4: RI-Rising Time; 5: PA-Positive affect; 6: NA-Negative affect; 7: PSQ- Perceived sleep quality; 8: SE- Sleep efficiency^

**Supplementary Table S5:** All path coefficients estimated from the structural model

|  | Original Est. | Bootstrap Mean | Bootstrap SD | T Stat. | 2.5% CI | 97.5% CI |
| --- | --- | --- | --- | --- | --- | --- |
| LEBA B1  ->  PT | -0.25 | -0.22 | 0.08 | -2.91 | -0.36 | -0.01 |
| LEBA B1  ->  MA | -0.08 | -0.09 | 0.09 | -0.90 | -0.25 | 0.09 |
| LEBA B1  ->  RT | -0.11 | -0.10 | 0.07 | -1.63 | -0.23 | 0.04 |
| LEBA B1  ->  RI | 0.02 | 0.02 | 0.06 | 0.40 | -0.09 | 0.14 |
| LEBA B1  ->  PA | -0.01 | -0.01 | 0.06 | -0.13 | -0.14 | 0.11 |
| LEBA B1  ->  NegA | 0.13 | 0.12 | 0.07 | 1.71 | -0.07 | 0.24 |
| LEBA B1  ->  PSQ | 0.00 | 0.01 | 0.06 | 0.05 | -0.12 | 0.13 |
| LEBA B1  ->  SE | 0.01 | 0.02 | 0.08 | 0.13 | -0.13 | 0.18 |
| LEBA B1  ->  Memory | -0.01 | -0.02 | 0.08 | -0.14 | -0.16 | 0.14 |
| LEBA B1  ->  Concentration | -0.08 | -0.07 | 0.06 | -1.33 | -0.18 | 0.04 |
| LEBA B2  ->  PT | 0.20 | 0.19 | 0.07 | 3.03 | 0.06 | 0.31 |
| LEBA B2  ->  MA | 0.08 | 0.08 | 0.06 | 1.39 | -0.04 | 0.20 |
| LEBA B2  ->  RT | 0.17 | 0.17 | 0.06 | 2.69 | 0.04 | 0.29 |
| LEBA B2  ->  RI | 0.14 | 0.13 | 0.06 | 2.22 | 0.01 | 0.25 |
| LEBA B2  ->  PA | 0.33 | 0.33 | 0.05 | 6.32 | 0.22 | 0.42 |
| LEBA B2  ->  NegA | 0.00 | 0.00 | 0.07 | -0.04 | -0.13 | 0.13 |
| LEBA B2  ->  PSQ | 0.07 | 0.07 | 0.07 | 1.10 | -0.05 | 0.21 |
| LEBA B2  ->  SE | -0.09 | -0.09 | 0.07 | -1.23 | -0.23 | 0.06 |
| LEBA B2  ->  Memory | -0.09 | -0.09 | 0.06 | -1.54 | -0.19 | 0.02 |
| LEBA B2  ->  Concentration | 0.04 | 0.03 | 0.07 | 0.54 | -0.10 | 0.16 |
| LEBA B3  ->  PT | -0.22 | -0.23 | 0.05 | -4.13 | -0.33 | -0.12 |
| LEBA B3  ->  MA | -0.12 | -0.12 | 0.06 | -2.09 | -0.23 | -0.01 |
| LEBA B3  ->  RT | -0.25 | -0.25 | 0.05 | -4.61 | -0.36 | -0.15 |
| LEBA B3  ->  RI | -0.23 | -0.24 | 0.06 | -3.96 | -0.35 | -0.12 |
| LEBA B3  ->  PA | 0.05 | 0.04 | 0.06 | 0.78 | -0.08 | 0.16 |
| LEBA B3  ->  NegA | 0.09 | 0.09 | 0.06 | 1.58 | -0.03 | 0.20 |
| LEBA B3  ->  PSQ | 0.13 | 0.13 | 0.06 | 2.21 | 0.01 | 0.24 |
| LEBA B3  ->  SE | -0.03 | -0.02 | 0.07 | -0.39 | -0.16 | 0.11 |
| LEBA B3  ->  Memory | 0.07 | 0.06 | 0.06 | 1.19 | -0.05 | 0.17 |
| LEBA B3  ->  Concentration | 0.09 | 0.09 | 0.06 | 1.56 | -0.03 | 0.21 |
| LEBA B4  ->  PT | -0.04 | -0.03 | 0.07 | -0.54 | -0.17 | 0.11 |
| LEBA B4  ->  MA | 0.02 | 0.01 | 0.08 | 0.21 | -0.13 | 0.16 |
| LEBA B4  ->  RT | -0.14 | -0.13 | 0.08 | -1.72 | -0.26 | 0.05 |
| LEBA B4  ->  RI | -0.05 | -0.06 | 0.08 | -0.59 | -0.20 | 0.11 |
| LEBA B4  ->  PA | -0.04 | -0.04 | 0.06 | -0.69 | -0.16 | 0.08 |
| LEBA B4  ->  NegA | -0.02 | -0.01 | 0.06 | -0.41 | -0.13 | 0.10 |
| LEBA B4  ->  PSQ | 0.05 | 0.05 | 0.06 | 0.83 | -0.08 | 0.17 |
| LEBA B4  ->  SE | -0.02 | -0.03 | 0.09 | -0.19 | -0.20 | 0.15 |
| LEBA B4  ->  Memory | 0.06 | 0.06 | 0.07 | 0.88 | -0.08 | 0.19 |
| LEBA B4  ->  Concentration | 0.05 | 0.04 | 0.06 | 0.77 | -0.08 | 0.15 |
| LEBA B5  ->  PT | 0.15 | 0.15 | 0.06 | 2.34 | 0.02 | 0.27 |
| LEBA B5  ->  MA | 0.14 | 0.14 | 0.07 | 2.02 | 0.00 | 0.27 |
| LEBA B5  ->  RT | 0.15 | 0.14 | 0.07 | 2.15 | 0.01 | 0.27 |
| LEBA B5  ->  RI | 0.09 | 0.10 | 0.07 | 1.25 | -0.04 | 0.23 |
| LEBA B5  ->  PA | 0.10 | 0.10 | 0.06 | 1.79 | -0.01 | 0.21 |
| LEBA B5  ->  NegA | 0.19 | 0.18 | 0.09 | 2.13 | -0.02 | 0.34 |
| LEBA B5  ->  PSQ | -0.16 | -0.15 | 0.06 | -2.57 | -0.27 | -0.03 |
| LEBA B5  ->  SE | -0.03 | -0.03 | 0.07 | -0.49 | -0.16 | 0.11 |
| LEBA B5  ->  Memory | 0.10 | 0.10 | 0.06 | 1.56 | -0.03 | 0.22 |
| LEBA B5  ->  Concentration | 0.01 | 0.01 | 0.06 | 0.19 | -0.11 | 0.13 |
| PT  ->  PSQ | -0.05 | -0.05 | 0.08 | -0.58 | -0.21 | 0.12 |
| PT  ->  SE | -0.02 | -0.01 | 0.08 | -0.21 | -0.18 | 0.15 |
| PT  ->  Memory | 0.00 | 0.00 | 0.07 | 0.05 | -0.15 | 0.14 |
| PT  ->  Concentration | -0.09 | -0.09 | 0.07 | -1.24 | -0.23 | 0.05 |
| MA  ->  PSQ | -0.21 | -0.20 | 0.06 | -3.43 | -0.32 | -0.09 |
| MA  ->  SE | 0.12 | 0.13 | 0.07 | 1.74 | -0.01 | 0.27 |
| MA  ->  Memory | -0.13 | -0.14 | 0.06 | -2.27 | -0.25 | -0.02 |
| MA  ->  Concentration | -0.11 | -0.11 | 0.06 | -2.05 | -0.22 | 0.00 |
| RT  ->  PSQ | 0.04 | 0.04 | 0.07 | 0.61 | -0.10 | 0.18 |
| RT  ->  SE | 0.00 | 0.00 | 0.08 | 0.04 | -0.16 | 0.16 |
| RT  ->  Memory | -0.11 | -0.11 | 0.07 | -1.53 | -0.24 | 0.03 |
| RT  ->  Concentration | 0.06 | 0.06 | 0.07 | 0.87 | -0.08 | 0.19 |
| RI  ->  PSQ | 0.02 | 0.03 | 0.05 | 0.41 | -0.08 | 0.14 |
| RI  ->  SE | 0.07 | 0.07 | 0.07 | 0.89 | -0.08 | 0.21 |
| RI  ->  Memory | 0.01 | 0.01 | 0.05 | 0.24 | -0.08 | 0.12 |
| RI  ->  Concentration | -0.05 | -0.05 | 0.05 | -0.92 | -0.15 | 0.06 |
| PA  ->  PSQ | -0.19 | -0.19 | 0.06 | -3.05 | -0.30 | -0.06 |
| PA  ->  SE | 0.21 | 0.21 | 0.07 | 3.00 | 0.07 | 0.34 |
| PA  ->  Memory | 0.05 | 0.05 | 0.06 | 0.75 | -0.08 | 0.17 |
| PA  ->  Concentration | -0.05 | -0.06 | 0.06 | -0.83 | -0.18 | 0.07 |
| NegA  ->  PSQ | 0.28 | 0.29 | 0.06 | 4.97 | 0.18 | 0.40 |
| NegA  ->  SE | -0.01 | -0.01 | 0.06 | -0.08 | -0.13 | 0.11 |
| NegA  ->  Memory | 0.37 | 0.36 | 0.06 | 6.30 | 0.25 | 0.47 |
| NegA  ->  Concentration | 0.32 | 0.31 | 0.06 | 5.75 | 0.20 | 0.42 |
| PSQ  ->  PA | -0.29 | -0.29 | 0.05 | -5.39 | -0.39 | -0.18 |
| PSQ  ->  NegA | 0.37 | 0.38 | 0.05 | 7.17 | 0.27 | 0.48 |
| PSQ  ->  Memory | 0.13 | 0.14 | 0.06 | 2.27 | 0.02 | 0.25 |
| PSQ  ->  Concentration | 0.22 | 0.23 | 0.06 | 3.87 | 0.11 | 0.34 |
| SE  ->  PA | 0.21 | 0.21 | 0.05 | 3.84 | 0.10 | 0.31 |
| SE  ->  NegA | -0.06 | -0.07 | 0.06 | -1.16 | -0.18 | 0.04 |
| SE  ->  Memory | -0.04 | -0.04 | 0.05 | -0.82 | -0.15 | 0.06 |
| SE  ->  Concentration | -0.07 | -0.07 | 0.05 | -1.32 | -0.17 | 0.03 |

^PT- Peak time; MA-Morning affect; RT-Retiring Time; RI-Rising Time; PA-Positive affect; NA-Negative affect; PSQ- Perceived sleep quality; SE- Sleep efficiency^
